# Teachers’ perspectives on barriers and motivators to physical activity participation in children from ethnic minority groups in Newcastle upon Tyne: A qualitative study

**DOI:** 10.1371/journal.pone.0342233

**Published:** 2026-02-05

**Authors:** Joyce Ene Omenyo Omojor-Oche, Gavin Daniel Tempest, Florentina Hettinga, Kandianos Emmanouil Sakalidis

**Affiliations:** 1 School of Sport Exercise and Rehabilitation, Faculty of Health and Wellbeing, Northumbria University, Newcastle upon Tyne, United Kingdom; 2 Department of Human Movement Sciences, Faculty of Behavioural and Human Movement Sciences, Vrije Universiteit, Amsterdam, The Netherlands; 3 School of Psychology, Faculty of Health and Wellbeing, Northumbria University, Newcastle upon Tyne, United Kingdom; Universitatea Transilvania din Brasov, ROMANIA

## Abstract

Physical inactivity among children in Western countries, especially children from ethnic minority groups, is a global health concern. Schools provide an ideal setting to address children’s physical activity needs, with teachers playing a major role. Therefore, the aim of this study was to explore teachers’ perspectives on barriers and motivators to physical activity participation in children from ethnic minority groups and to determine through their suggestions, how schools can be better supported to provide a physical activity-enabling environment. A purposive sample of eight primary school teachers in the Northeast of England, United Kingdom, participated in semi-structured interviews conducted through a combination of face-to-face and virtual settings. Reflexive thematic analysis identified seven barriers and nine motivators to physical activity, distributed across different levels of the socio-ecological theory. At the intrapersonal level, motivators included fun and wellbeing, whereas barriers included behavioural issues, and increased screen time. The interpersonal level encompassed influences of friends, parents, and teachers. At the institutional level, in-school activities were identified as motivators. At the community level, environmental influence and perceptions of safety were identified as barriers. At the public policy level, government/local council and resources were identified as both barriers and motivators. Notably, the teachers’ perspectives align with previous findings on barriers and motivators to physical activity among children from ethnic minority groups in the Northeast of England, while contributing to policy-level insights. These policy-level insights highlight the importance of staff training, government funding, and sports infrastructure improvements.

## 1. Introduction

The World Health Organization (WHO) recommends that children aged 5–17 years participate in an average of 60 minutes of moderate-to-vigorous intensity physical activity (PA) per day [1]. Complementing this, the United Kingdom (UK) government recommends that children should have access to (at least) 30 minutes of PA during school hours and (at least) 30 minutes outside of school [2]. Despite these clear guidelines, physical inactivity among children remains a persistent global health burden [3]. In England, only 44.6% of children aged 5–16 years meet the WHO’s 60-minute daily PA guidelines [4]. This problem is particularly acute among children from ethnic minority backgrounds [5], especially those from lower economic households [6].

Schools can play a crucial role in increasing PA levels in children and adolescents [7], potentially addressing barriers encountered at home while providing facilitators such as sports equipment-factors identified as particularly important for children from ethnic minority backgrounds [8]. Research demonstrates that children acquire higher PA levels in school environments compared to home during the holidays [9]. Moreover, school-based PA, including recess and activity breaks, correlates positively with educational outcomes and behaviour [10], as well as with enhanced mental, physical and cognitive development [11].

Addressing PA behaviour among children from ethnic minority backgrounds is critical for mitigating health inequalities, obesity and sedentary lifestyles as they go through adolescence and into adulthood [12]. According to Allender et al. [13], early exposure to PA is crucial for children’s development as well as long-term PA participation. Data from a cross-sectional study conducted on primary schools within Europe suggest that most children spend 65% of their time in school on sedentary activities, especially children from ethnic minority groups from low- and mid-income neighbourhoods [14]. Within the school setting, several structural barriers impede PA participation including insufficient leadership support and a crowded curriculum that prioritises academic achievement [15]. While a lack of variety of sports and organised team sports were identified by children from ethnic minority groups as barriers to PA participation [16], the recruitment of teachers who lack intercultural knowledge of ethnic minority groups, makes it more difficult to address structural inequalities inherent in PA participation among this population [17].

Challenges such as a lack of variety of sports can significantly impede the achievement of optimal PA levels in children from ethnic minority groups [18]. The school environment should serve as an enabling setting for all children to engage in moderate to vigorous PA, by providing opportunity, accessible spaces (spaces to play), social support and appropriate equipment [19]. Schoolteachers bear significant responsibility for stimulating PA through teaching, coaching and motivating the children [20]. They typically determine the type of activities (PA-focused) and how long the children spend engaged in them [21]. Given that children from ethnic minority backgrounds experience higher levels of physical inactivity and face more challenges at achieving PA at home [22], tailored approaches are needed to engage them effectively within the school setting. Previous studies including a children’s qualitative study [16] have already explored barriers and motivators to PA participation in children from ethnic minority groups in Newcastle upon Tyne, by collecting qualitative data directly from children about their perceived barriers and motivators. This present study extends this work by exploring teachers’ perspectives on the barriers and motivators to PA participation among children from ethnic minority groups living in Newcastle upon Tyne, in the North-East of England and to determine through their suggestions, how schools can be better supported to provide a PA enabling environment for children, especially, children from ethnic minority groups.

## 2. Materials and methods

### 2.1. Participants

Participants were recruited from primary schools in Newcastle upon Tyne in the North-East of England specifically from areas characterised as economically disadvantaged with estimated annual household incomes of £52,500 [23]. In determining ethnic group classifications, we adopted the UK government’s recommended terminology “ethnic minority groups” rather than “BAME” (Black, Asian and Minority Ethnic) as the latter term has been identified as non-inclusive, failing to encompass groups such as those of mixed heritage [24]. For this study, the purposive sampling strategy, which focuses on participant selection with an emphasis on the study’s objectives, was employed [25]. Primary schools with diverse ethnic representation were initially approached with a letter of introduction and comprehensive study information. Interested schools and teachers were subsequently contacted by the first author to schedule interviews with information and consent forms provided in advance.

#### 2.1.1. Sample characteristics.

The recruitment strategy yielded eight participants aged between 23 and 61 years, comprising: one head teacher, two physical education (PE) teachers, three classroom teachers, and two teaching assistants. All participants worked in primary schools attended by children from both ethnic minority and White British backgrounds. This diverse sampling approach was deliberately employed to ensure methodological rigour. By collecting data from teachers with varied characteristics, including different ethnicities, gender, (one male and seven female participants), and teaching roles, we ensured that findings were comprehensive and reflected multiple perspectives [26].

To ensure anonymity, unique codes were assigned to both schools and participants, guaranteeing that all collected and stored data contained no personal identifiers that could reveal participant or school identities. Demographic details of the participants are provided in Table 1 below.

**Table 1:** Demographic information of schools and participants.

Participant ID	School	Role	Gender	Age (years)	Ethnicity
PT01	1	PE teacher	Female	38	White British
PT02	1	Class teacher	Female	36	White British
PT03	2	PE teacher	Female	35	White British
PT04	2	Head teacher	Female	49	White British
PT05	5	Teaching Assistant	Female	27	Black African
PT06	5	PE Teacher	Male	30	Black African
PT07	6	Teaching Assistant	Female	23	Black African
PT08	7	Class Teacher	Female	61	White British

Participants were eligible for inclusion if they: (1) were currently employed as a primary school teacher in the UK; (2) demonstrated proficiency in English; and (3) provided informed consent. A total of eight teachers from five primary schools participated in this study. These five schools collectively enrolled 1,516 children; with the proportion of children whose first language was not English ranging from 18.8% to 60%. All data collection was conducted by the first author (JO). Recruitment and data collection stopped once saturation was reached, as further interviews were not adding new insights relevant to the research questions.

### 2.2. Ethical approval

Ethical approval (3270) was granted by the School of Sport, Exercise and Rehabilitation Research Ethics Committee of Northumbria University.

### 2.3. Procedure

Semi-structured interviews were conducted in two stages: The first set of interviews was conducted from 21/03/2023 to 02/06/2023. Following the recommendation of the co-authors, a second set of interviews was conducted from 18/07/2024 to 22/07/2024. The interviews were conducted on a one-to-one basis in a face-to-face context (two teachers) and via video calling platforms (six teachers). The face-to-face interviews were conducted in the schools where the teachers taught, while the online interviews were conducted at locations where the teachers were most comfortable. The semi-structured approach was employed for in-depth exploration of the research question, as participants are more likely to relax and open up about topics [27]. Interview questions were developed in line with the study’s aim and objectives and addressed topics such as participants’ perceptions and opinions on what might be the barriers or motivators to PA participation among children from ethnic minority groups and what types of PA children from ethnic minority groups enjoy. Further probing regarding children’s PA participation within and outside of school, and teachers’ personal thoughts on allotted activity time within schools, were employed to obtain richer data. Participants were also encouraged to make suggestions for an optimal PA environment and to discuss the role of teachers in addressing the barriers and motivators to PA participation among children from ethnic minority groups. The questions were posed by the first author flexibly using the interview guide, allowing for a free flow of discussion.

The interviews were conducted in English by the first author and recorded on Teams for online interviews and an audio recorder for the face-to-face interviews (a Sony ICD model PX240 recording device). The semi-structured interviews lasted between 45 and 60 minutes per teacher.

### 2.4. Analysis

All eight semi-structured interviews were transcribed verbatim using the Otter.AI online software transcription tool. The first author (JO) checked the transcribed data against the recordings for accuracy. Transcripts were analysed using reflexive thematic analysis [28].

Thematic analysis places emphasis on the identification, analysis, and interpretation of data, thereby presenting a rigorous and auditable process that can easily be replicated [29]. This study was underpinned by the constructivist paradigm. That means the authors understood that people construct knowledge through their own interactions with the world. Thus, the perceptions of participants were interpreted as their personal experiences as teachers, while at the same time, the researchers considered the political, cultural, and historical context in which these experiences occurred [30]. The first author (JO) immersed and familiarised herself with the data by listening to the audio recording, checking against the transcription, and identifying patterns that generated the initial codes. The eight transcripts were exported to the NVivo software application (version 12) where the process of coding and derivation of subthemes and themes was undertaken.

The coding process and generated themes were discussed by the first author (JO) and last author (KS) who also coded the transcripts. To ensure fidelity, KS acted as a “critical friend” questioning JO’s assumptions, coding process and generated themes [31]. This was to promote reflection and bolster the further development of relevant themes [32]. JO and KS met afterwards for a review of potential themes and subthemes. Participants’ responses, familiar patterns, and the themes they represented were deliberated upon by JO and KS, and differences and similarities were resolved in line with the study aim and within the context of the teachers’ responses. Afterwards, JO and KS deliberated on the agreed themes and subthemes and what they meant. Final themes and subthemes were discussed by all four authors. These processes were followed to ensure rigour in the inductive thematic development process [33].

## 3. Results

### 3.1. Themes

A total of seven barriers and nine motivators were inductively identified in this study (Table 2).

**Table 2:** Themes: Barriers and motivators.

Barriers/Motivators	Themes
Barriers	Behavioural issues
	Screen time
	Social environment in school
	Parents
	Environmental obstacles
	Inadequate resources
	Local authority
Motivators	Fun and wellbeing
	Social circle
	Teachers
	Introduction of physical activity breaks
	Introduction of a variety of sports
	School environment
	Environmental influences
	Resources
	Government

Although the analysis was conducted inductively, the identified themes clearly mapped to the different levels of socio-ecological theory and are thus presented below in the context of this theory. The socio-ecological theory posits that a person’s behaviour is influenced by factors at multiple levels including the intrapersonal, interpersonal, organisational (institutional), community and policy levels [34].

**Fig 1:**
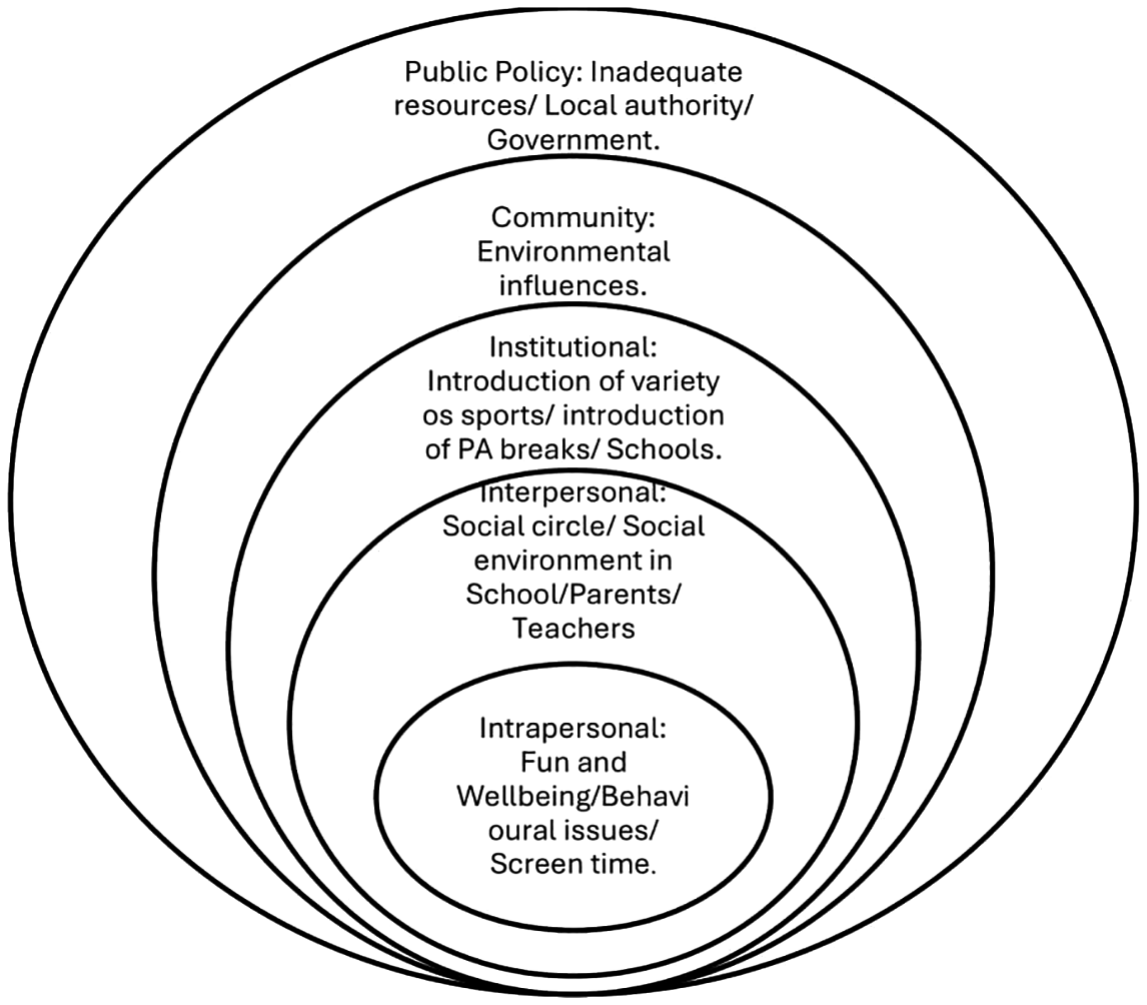
Socio-ecological representation of themes.

The identified themes show each level and how they impact the PA participation of children from ethnic minority groups (Fig 1). At the intrapersonal level, motivators identified include fun and wellbeing, while barriers identified include behavioural issues (e.g., age-related barriers, post-COVID impact) and screen time. At the interpersonal level, motivators and barriers identified include the social circle (friends, parents) and teachers. At the institutional level, barriers and motivators identified were based in school and they include the introduction of physical activity breaks and the introduction of a variety of sports. At the community level, environmental influences/obstacles were identified as barriers and motivators. At the public policy level, the local council with responsibility of funding for resources such as sports equipment, providing playing grounds, etc. was identified as a barrier. On the other hand, some of the teachers reported the availability of government funding for sports resources as a motivator to PA participation in children from ethnic minority groups.

### 3.2. Barriers

#### 3.2.1. Behavioural issues.

Behavioural issues were reported by some of the teachers as a barrier to PA participation among children, and this made PA supervision difficult. Some of the teachers suggested that the negative behaviour and attitude exhibited by some of the children (PT02, class teacher; PT05, teaching assistant) was a huge barrier to the uptake of PA as it prevented different year groups from playing together (PT02, class teacher). On the other hand, some of the teachers suggested that as children got older (PT01, PE teacher; PT02, class teachers) their PA behaviour sometimes got worse, thereby making it difficult to motivate the children to be physically active or putting the teachers off trying to encourage them to be active:

In terms of physical activities, I think maybe if other kids are, I will not say bully because it’s just kids being kids. Maybe they tell them “Yeah, you’re meant to be strong and you’re black. You’re meant to be stronger” and things like that. So that’s the only thing I can see in terms of barriers for them (PT05, teaching assistant).

#### 3.2.2. Screen time.

Some of the teachers (PT02, PT08 class teachers; PT03, PE teacher) pointed out the children’s excessive use of technology, as a barrier to uptake of PA; ‘a lot of the time, a lot of children are spending more time on computer games, rather than being outside’ (PT03, PE teacher), thus leading to a sedentary lifestyle and being physically inactive.

They just seem to just want to be on their tablets and then we say to them all the time, like, playing games literally kills your brain cells… and it’s not good for you, but they’re just not bothered because they just want to play games inside (PT02, class teacher).

#### 3.2.3. Social environment in school.

Children’s social environment plays very crucial role in PA participation especially for children from ethnic minority groups. Factors such as peer pressure (PT01, PE teacher) untrained or inadequate staff to supervise could be a barrier to PA participation in children from ethnic minority groups:

But again, then you need staff to supervise that, and again, we haven’t got it. That’s another issue with like break and lunchtimes is there’s just not enough staff to supervise. And the people that we’ve got outside, obviously, like do their best there at lunchtime, but they’re not teachers. And they’re not like they’re not trained as teachers (PT02, class teacher).

#### 3.2.4. Parents.

Parental barriers such parents’ perception of safety and their professional expectations for their children could be a barrier to PA participation in children from ethnic minority groups.

I don’t think it’s as safe to be out and about at nighttime as it used to be years ago. So, a lot of parents I think, are probably concerned about letting their children be out and about on at nighttime (PT03, head teacher).

And some of these ethnic minority parents, you know, they want their kids to be doctors, they want their kids to be lawyers. If you tell them anything about sports, they will say, ‘go and read your books’ (PT06, PE teacher).

#### 3.2.5. Environmental obstacles.

Environmental obstacles such as bad weather, rough playing ground or a lack of playgrounds at home or at school are significant barriers to PA participation among children from ethnic minority groups. Some of the teachers (PT01 and PT03, PE teachers; PT04, head teacher) reported the lack of structural sporting facilities or sports facilities such as playgrounds needing repairs as a contributing factor to lower PA levels: ‘We don’t have a lot of flat ground where children can play’ (PT04, head teacher).

I feel like something that might stop them from being physically active could be like home environment. Not really having like, you know, maybe like a I’m just like maybe assuming, but maybe having like a not having like a spacious place to do running it and playing and you know how being able to be that creative (PT07, teaching assistant).

#### 3.2.6. Inadequate resources.

All the teachers identified resources such as insufficient PE/play time, lack of free and accessible youth community centres, and a lack of funds for the purchase of sports equipment as barriers to PA participation among children from ethnic minority groups: ‘I don’t think there’s a great deal of community sports for children that are free, that are accessible to everybody’ (PT02, class teacher); ‘And then I guess for parents, it’s not just time but it’s also money. So, we can provide things for free in school. But they’re not going to be free when you’re outside of school’ (PT01, PE teacher).

#### 3.2.7. Local authority.

In the course of performing their functions and roles, the local authority or local council allegedly closed some community centres, parks and recreation centres making it difficult for children and youths, already facing barriers of unsafe neighbourhoods and lack of play gardens at home, to participate in PA (PT03, PE teacher; PT04, head teacher). ‘So, the local authority got rid of swimming pools; that seems silly, whereas our community used to use that facility quite a lot’ (PT04, head teacher).

### 3.3. Motivators

#### 3.3.1. Fun and wellbeing.

The participants reported the positive impact of playing outdoors on children. One of the PE teachers (PT01) reported how playing outdoors was good for children’s wellbeing, especially as they could play with other children. Also, PA was reported to improve overall learning and prevent some of the children from being disadvantaged in certain situations’ (PT07, teaching assistant) and the head teacher (PT04) reported how children playing outside has been instrumental in motivating them to be physically active, especially after COVID. Furthermore, PA was reported as an instrument for positive behaviour in children:

Yeah, exactly. They tend to become calmer and all that. It tends to feel relaxed. ok, how do I explain it? Their energy tends to be very low because they put the energy in sports and something beneficial to them (PT05, teaching assistant).

#### 3.3.2. Social circle.

Participants reported the positive impact that a child’s social circle, such as their parents, friends, and peer-led PA, can have on children’s engagement in PA (PT03, PE teacher; PT04, head teacher). Some children were reported as looking forward to play time because it would include their friends as they like to do things with their friends (PT04, head teacher), while more formal peer support schemes were also thought to be helpful:

So, the playground crew led by the children themselves; they come up with their own games, two of those people [children] are on the school council, so they speak to the rest of the school about what kinds of things they would like to see (PT03, PE teacher).

#### 3.3.3. Teachers.

Teachers and sports coaches play very pivotal roles in children’s PA uptake. Some of the teachers reported the importance of visiting coaches ‘I think within schools, they’re always motivated when we get visiting coaches to take lessons rather than (Referring to herself) Mrs.****** (PT08, class teacher). While one of the participants noted that having a PE teacher who understands the PA needs of the children is important:

So yeah, so I do a girl’s club where, because some girls like want to try games, and they feel intimidated by the boys as they get older. Yeah, so they come, and they just have a pick. So, we do badminton, or volley. So, I know one girl (referring to an Asian girl) mentioned volleyball. So, we do volleyball as well. So, there’s, there’s plenty of options (PT01, PE teacher).

#### 3.3.4. Introduction of physical activity breaks.

Introduction of PA breaks was reported by the PE teachers (PT01 & PT03); as a PA alternative; ‘we try to keep the children active not just in PE or in lunch or break. But during the class lessons, we do active Maths, we do just dance. If we can get them outside, we will ‘(PT01, PE teacher), and beneficial to other curriculum (subjects):

I think the only other thing is kind of in my opinion, I’ve seen the benefits of physical activity on other areas of the curriculum. So, quite often, if it’s an English lesson, and it’s a hot day, and the children are getting tired, a quick physical brain break really helps them to focus again. So, it does have a massive impact on all areas, not just health and fitness’ (PT03, PE teacher).

#### 3.3.5. Introduction of a variety of sports.

The introduction of a variety of sports, team sports such as football and the availability of indoor sports during bad weather were some of the factors reported as motivators to uptake of PA in children (PT01, PT03 and PT06, PE teachers; PT02, class teacher; PT07, teaching assistant). Also, for girls who might be shy or not want to play with the boys as a result of cultural differences, introducing an all-girls club might motivate them to be physically active (PT01, PE teacher).

Yeah, I think maybe just like having a different activity, something more exciting for them to do. So, things like having like the climbing wall was something different. And it’s a very different kind of physical activity during the climbing wall and it is very difficult

(PT02, class teacher).

#### 3.3.6. School environment.

The school environment was reported by some of the participants as crucial in the uptake of PA in children from ethnic minority groups. Some of the schools provided after school clubs, PE lessons, and summer activities’ (PT04, head teacher). In fact, children from ethnic minority groups were reported as being more active than children from ethnic minority children by a few of the teachers (PT05 and PT07, teaching assistants, PT06, PE teacher, PT08, class teacher):

I will say like from what I’ve seen that I think minority students and children did exceed athletically above their peers compared to like other ethnicities like the African, the Asian. I would say those excelled much higher than their peers. You may have been British or and yeah, definitely like there was an example of one of the pupils at the school. He was black and he was known as like the fastest boy in that year or in that class (PT07, teaching assistant).

#### 3.3.7. Environmental influences.

Factors such as favourable weather conditions (PT01, PE teacher), adequate safe play space and a suitable playground (PT04, head teacher) were factors reported by some of the participants as facilitators for the uptake of PA in children from ethnic minority groups. Particularly for families who do not have play space or gardens, utilising the school or a well-equipped general playground was reported to be a motivator for uptake of PA (PT04, head teacher).

You might see things like football, they can’t do it on the concrete or, you know, certain things like cartwheels or, you know, just stuff that they would maybe have more variety of doing compared to the concrete. It’s a bit limited, whereas on the field you know it’s unlikely for them to hurt themselves, you know, with other things. If it’s on grass and stuff (PT07, teaching assistant).

#### 3.3.8. Resources.

Participants reported free after-school clubs (PT02, class teacher; PT04, head teacher), increased allotted playing times, and funding for sporting equipment (PT01, PE teacher; PT02, class teacher) as factors that would positively impact uptake of PA in children: ‘At the minute, we don’t have a problem funding equipment, but that could be a barrier in the future. But I know for the next two years, the government is providing funding, which helps us buy equipment’ (PT01, PE teacher).

I have nieces and nephews in the UK here and their parents are currently paying for extracurricular activities. The mum even drives as far from XXX to XXX to take him to his sporting events and everything, but I think most Nigerian parents, that are not settled, they will not have the time for that (PT05, teaching assistant).

#### 3.3.9. Government.

The government plays an important role in the uptake of PA as it provides the schools with the needed funding and sports equipment (PT01, PE teacher; PT02, class teacher) and has the power to close community youth centres and provide safe playgrounds for children (PT04, head teacher).

I think it’s, like, money. Because there’s not enough. I know we get sports funding. But then if you wanted to get, like, a huge range of… activities that would… cost a small fortune, doesn’t it… to get even things like tennis nets and stuff like that, like, they’re really expensive. So, it may be more, more help, but from my point of view (PT02, class teacher).

### 3.4. Suggestions from teachers

Outside of the themes pertaining to barriers and motivators, but important to consider going forward, were the suggestions offered by teachers on how schools can be better supported in their PA provision. There was a focus on funding for equipment, including the mention that greater funding would allow the provision of greater varieties of sport. Funding would also support the upkeep of school facilities such as playgrounds. Having access to schoolyards and fields was also noted as a suggestion, as some schools need their pupils to travel to other locations such as sports centres for their PA sessions. Beyond facilities and funding, one of the participants spoke about the need for training:

And I would say that probably most teachers often feel that there are elements of the PE curriculum because it is so, so broad that we just are reticent to teach because we’re not; we don’t feel we know enough about it. And I would say one of the particular ones often is the outdoor and adventurous activities section of the curriculum, which is supposed to include things like auto sort of leads up to orienteering and that type of activity (PT08, class teacher).

## 4. Discussion

The aim of this study was to explore teachers’ perspectives on barriers and motivators to PA participation among children from ethnic minority groups and to determine, through suggestions, how schools can be better supported to provide a PA-enabling environment for children from ethnic minority groups. Findings from this study suggest barriers and motivators to PA participation in children from ethnic minority groups occur at different levels beginning from the individuals themselves to external influence beyond the child’s control. The barriers to PA participation among children from ethnic minority groups identified by the teachers through an inductive analytical method included behavioural issues, screen time, social circle, parents, teachers, environmental obstacles, inadequate resources and the local authority. On the other hand, motivators included fun and wellbeing, social circle, teachers, the introduction of physical activity breaks, the introduction of a variety of sports, schools, environmental influences, resources, and the government. The identified barriers and motivators can be better understood using the socio-ecological theory [35,36].

The socio-ecological theory provides a theoretical understanding of PA behaviour in children [36]. For example, it identifies the different levels or factors that could impact the uptake of PA in children, including children from ethnic minority groups, and this includes individual characteristics (e.g., beliefs, attitude, gender), the social environment (parents, friends), the physical environment (equipment, sports facilities) and government policies, funding, and provision of sporting equipment [37]. Understanding the PA behaviour of children from ethnic minority groups and/or developing a PA intervention purposed to address health inequality could therefore be guided by the socio-ecological model [38]. We would like to use this theory as the theoretical basis to explain the results, as well as a guide for future studies.

At the intrapersonal level, several behavioural issues were reported by the teachers as a barrier to PA participation in children from ethnic minority groups. Some of the behavioural barriers were linked to peer pressure (social circle barriers) while others were linked to increased screen time. Other behavioural issues were linked to negative feedback with regards to race and performance. These behavioural issues make it difficult for some of the teachers to effectively supervise the children during PA, and some of the teachers even cancel PE activities. Some of the teachers attributed these behavioural issues to the detrimental impact of COVID-19, tying together the themes of behavioural issues and screen time [39]. The March 2020 COVID-19 pandemic led to precautionary measures, including the closure of schools, social distancing and staying at home [40]. Some of the teachers suggested that an increase in screen time was a harmful impact of the COVID-19 pandemic, and evidence prior to the pandemic noted that improved technology and excessive computer usage led to increased screen time viewing on laptops, televisions, phones, and electronic tablets, and was a risk factor for high levels of physical inactivity [41]. In contrast, other children (mostly White British children) demonstrated self-autonomy and self-confidence and considered PA to be enjoyable and beneficial to their mental health and wellness [16]. The teachers also reported playing outdoors as a huge motivating factor for children, especially from ethnic minority groups. Play was associated with fun (motivator), and consequently wellbeing and positive behaviour, especially if it was with friends [16], thus progressing from the intrapersonal to interpersonal level of the socio-ecological theory. In conducting any form of health promotion, there is a need to engage all stakeholders for an inclusive outcome [42]. This is because a previous qualitative study conducted with children from one school in Newcastle upon Tyne to determine their perceptions of barriers and motivators to PA participation [16], also reported as a behavioural issue ‘negative feedback from teammates’. Such feedback can negatively affect the PA experience of some of the children, thus making them lose interest in PA. At the interpersonal level of influence, the home front was reported as a barrier in the form of a lack of parental PA support or involvement. This barrier was also identified in the qualitative children study [16]; the children from ethnic minority groups reported parents as barriers as the parents would not let them go out to play [16]. The teachers in this study also reported that parents did not let their children out to play, but unlike the children’s study, this was linked to safety issues or fear of gang-related activities. The present study reports parents as barriers because of their professional expectation or may even overburden them with household chores. In contrast, parents, siblings, friends and family members were reported by some of the children from the white British groups, as motivating factors in PA participation [16]. This emphasizes the critical role that parents/guardians play in children participating in PA, suggesting a need to create more awareness among parents from ethnic minority groups on the need to support their children in PA activities.

An aversion to supervising PA activities due to limited PA knowledge was reported by some of the teachers as a barrier to PA participation. In contrast, children from ethnic minority groups reported the positive role their PE teacher played in motivating them to participate in PA by reorganising the team sports and motivating them to be kind to one another [16]. This barrier could be addressed through appropriate staff training, as suggested by one of the participants when asked how schools could be better supported in their PA provision [43]. In addition to training, staff members should be educated on the importance of PA [44]. This brings to the fore Ajzen’s theory of planned behaviour [45], which suggests a person’s behaviour is influenced by their intention and control; intentions (in this case to teach PA) are influenced by the subjective norm, attitude, and perceived behavioural control relating to the behavioural issues exhibited by the children [46]. Available evidence suggests a correlation between perceived behavioural control and attitude on intention [47] and that professional or additional training using the theory of planned behaviour framework predicts inclusive training practice in teachers, thus improving intention and behaviour towards inclusive education in this case, PA education [48]. As the themes in this current study suggest, teachers play crucial roles as both motivators and potential barriers to the uptake and continuance of PA behaviour in children [49], there is therefore a need to positively impact their attitude [50], and this can be done through the recruitment, training and engagement of the teachers especially with regards to managing the behaviours of children and positively nudging them towards the uptake of PA [51]. In addition to addressing teachers’ attitudes, there is a need to address subjective norms and perceived behavioural control. This can be achieved by creating an environment within the school in which staff support ethnically diverse children with PA, which will in turn create positive norms around PA. Furthermore, the training provided should be aimed at increasing teachers’ self-efficacy for the delivery of PA sessions, thereby improving their perceived behavioural control [52].

The next level of influence is the institutional level, and this includes the schools where the children spend most of their waking hours. The introduction of a variety of sports and the structuring of sports activities, along with free after-school clubs, were mentioned as very important factors for PA participation in children from ethnic minority groups in the qualitative study [16]. School activities such as playtime and PE address the deficit in PA levels in less active children, especially in children from lower socio-economic groups and those from ethnic minority groups who may lack safe neighbourhoods for walking or free, accessible recreational facilities and parks [53].

Influences on PA at the next level of the socio-ecological theory, are the community or environmental level. These include factors such as weather, homes lacking community playing areas, safe walking, and bike paths. The impact of the environment such as suitable walking or playing grounds, and the lack of playing areas at home were also reported by the teachers as factors influencing children’s PA participation. Similarly, some of the children in the qualitative study reported the lack of playing areas at home, which made participation in PA difficult [16]. Furthermore, at the environmental level, perception of safety was reported as a barrier to PA participation in this current study. However, perception of safety was not reported in the qualitative study of children, this could be due to their age as they were aged 8–11 years [16]. However, this factor plays a very crucial role in PA participation in children from ethnic minority groups, especially those from lower socio-economic status [5].

The environmental level closely relates with the policy level as factors such as the provision of PA infrastructures in schools and communities are a part of government responsibilities, thus suggesting a need for multilevel collaboration at all the levels especially between schools and the government. This cannot be overemphasised in the promotion of PA for children, as the government not only issues guiding policies but also determines the allotted play and PE time and provides the funding needed for sports equipment, among other things [54]. Policies necessitating time and resources for PE and PA in schools, as well as community structures that make being physically active accessible and convenient, are important factors in making schools and communities healthier settings [55]. Furthermore, the introduction of free, safe and accessible community centres and free after-school clubs as reported in this and a previous study [16], will go a long way in allaying the fears of parents and addressing the perception of safety barriers, while at the same time addressing the inadequate resource barrier (finance and time) they face [54]. However, government policies and the provision of resources alone will not address concerns about physical inactivity. Individual characteristics that influence PA decisions, such as the religious and socio-cultural backgrounds of ethnic minority groups must be considered in efforts to engage children in physical education programmes that promote active lives [56].

If we are to actively address inequality, diversity, and inclusivity, there is a need to bridge the persistent gaps between a largely White profession (teachers) and ethnically diverse school populations as one size might not fit all [57]. What children do and learn in PE classes and how they engage in PA are determined by the learning climate created and established by the teachers [55]. Furthermore, addressing the PA needs of children from ethnic minority groups that occur at the various levels of impact, as reported in the children’s qualitative study [16] and in the present study will go a long way in addressing the gaps in PA levels in children from ethnic minority groups [43]. In addition, addressing teacher-identified needs and gaps can improve working conditions and reduce existing PA inequities as well as address inequalities that children from ethnic minority groups, might experience in participating in PA; this is because teachers play very crucial roles, as evidenced from this study and other studies [58]. As such, there is a need to explore in depth, government’s perception of PA barriers and motivators among children from ethnic minority group, as well as seek ways to increase and improve PA-focused training for all teachers with a focus on ethnically diverse populations, to improve the PA experience for all children and especially children from ethnic minority groups [24]. This would improve teachers’ confidence in PA planning and delivery, resulting in better implementation and thereby improved student outcomes as well as addressing gaps in PA levels [51].

### 4.1. Strengths and limitations

In addition to previous children’s qualitative studies [16], this teacher-focused study provides policy-level insights on the barriers and motivators to PA participation among children from ethnic minority groups, thus providing valuable insights for developing targeted interventions to enhance PA participation for children from ethnic minority groups. It is also important to note the potential value of stakeholder-led interventions such as child-led PA (a subtheme), as reported by one of the teachers and the positive impact it has had on PA levels among children from ethnic minority groups. A limitation in this study is that only one male participant took part in the study. However, the sample aligns with the gender proportion of schoolteachers in the United Kingdom as 75.5% of schoolteachers in the UK are women [59].

### 4.2. Conclusion

Barriers to PA participation in children from ethnic minority groups identified by the teachers in this study included behavioural issues, too much screen time, children’s social circle, teachers, environmental obstacles, the low perception of safety, inadequate resources and the local authority. The motivators identified were fun and wellbeing, children’s social circle, teachers, the introduction of physical activity breaks, the introduction of a variety of sports, schools, positive environmental influences, available resources and the government. The barriers and motivators identified by teachers in the present study are similar to those identified by children in previous research in the North-East of England, except for the policy-level insights. Policy-level insights identified by the teachers suggest that staff training, funding for sports equipment, and the provision of sports infrastructure are some of the conditions that can enhance the PA-related duties of school staff. Although including children in intervention designs is important, engaging diverse stakeholders including the government, provides a more comprehensive perspective. Enhancing school-based PA is of particular importance for children from ethnic minority groups. This is because they engage in less PA outside of school due to the different barriers they confront compared to children from White British groups, such as parents fearing gang-related activities and not letting their children out to play, not having enough resources to pay for after-school clubs, and/or living in homes without playing grounds.

## Supporting information

S1 File S2 Inclusivity in global research questionnaire. (DOCX)
